# On the Role of Interoception in Body and Object Perception: A Multisensory-Integration Account

**DOI:** 10.1177/17456916221096138

**Published:** 2022-08-22

**Authors:** Wladimir Kirsch, Wilfried Kunde

**Affiliations:** Department of Psychology, University of Würzburg

**Keywords:** action-specific perception, body perception, embodied perception, intentional binding, multisensory integration, multisensory perception, object perception, perception and action, rubber-hand illusion, temporal binding

## Abstract

Various “embodied perception” phenomena suggest that what people sense of their body shapes what they perceive of the environment and that what they perceive of the environment shapes what they perceive of their bodies. For example, an observer’s own hand can be felt where a fake hand is seen, events produced by own body movements seem to occur earlier than they did, and feeling a heavy weight at an observer’s back may prompt hills to look steeper. Here we argue that such and various other phenomena are instances of multisensory integration of interoceptive signals from the body and exteroceptive signals from the environment. This overarching view provides a mechanistic description of what embodiment in perception means and how it works. It suggests new research questions while questioning a special role of the body itself and various phenomenon-specific explanations in terms of ownership, agency, or action-related scaling of visual information.

The (motor) theory is so simple and so easy to present that every one is glad to believe it. The only question that anyone cares to raise is how much of it will the known facts permit one to accept.

—Pillsbury (1911, p. 84)

The body of biological observers determines their perception. This is true for obvious reasons. For one, the sensors that mediate perception, such as eyes or skin receptors, are part of the body. Shifting the gaze or moving the hand from one place to another, for example, changes what people see or feel. Thus, observers determine which aspect of the world stimulates them. However, research in the last decades has revealed much more intriguing influences of body states on perception. For example, observers judge their covered own hand to be closer to a fake hand when a seen touch or movement of this fake hand corresponds to the felt touch or movement of their own body (“proprioceptive drift in the so called rubber hand illusion,” Botvinick & Cohen, 1998; Kalckert & Ehrsson, 2012). Events are perceived to occur earlier in time when they were produced by a body movement rather than when produced by another cause (“temporal binding,” Haggard et al., 2002). Characteristics of an observer’s body, such as its height, potential to move, or metabolic state, affect visual judgments, such as of the distance or size of environmental objects (often discussed under the umbrella of “action specific perception”; Proffitt & Linkenauger, 2013; Witt, 2011a).

All these phenomena and various others discussed later have in common that perceivers’ bodies somehow shape their perception. These phenomena can thus be summarized under the term “embodied perception.”^1^ Despite such a label, these phenomena have been studied in independent research branches and are explained in phenomenon-specific ways. An overarching mechanistic model of embodied perception is missing. Moreover, these phenomenon-specific explanations often come with a considerable theoretical overhead that is not well supported. For example, the rubber-hand illusion is commonly supposed to reflect “ownership” of the fake hand (i.e., an external object is assumed to become an integral part of own body; for reviews, see e.g., Braun et al., 2018; Kilteni et al., 2015; Riemer et al., 2019; Tsakiris, 2010). Temporal binding is supposed to be an implicit measure of agency over external events and thus sometimes called “intentional binding” (e.g., Haggard, 2005, 2017; Haggard & Tsakiris, 2009). Action-specific effects on perception putatively result from scaling of information about objects by the ability to interact with them (Proffitt & Linkenauger, 2013; see also e.g., Proffitt, 2013; Witt, 2011a).

Here, we scrutinize what “embodied” perception means. We suggest that all these phenomena can be termed “embodied” to the extent that perceptual input from the material body (sensed through so-called interoception; see e.g., Craig, 2002; Goldman, 2012) interacts with perceptual input from the body-external environment (exteroception). We thus trace back these superficially different phenomena to a common mechanistic ground. By doing so, we critically reflect and eventually refute the theoretical overhead that is inherent of various phenomenon-specific explanations.

## Basic Idea, Scope of Application, and Outline

The general idea behind the present proposal is rather simple: Interoceptive (e.g., proprioceptive) events enrich the perception of exteroceptive (e.g., visual) events and vice versa, according to tried-and-tested principles of multisensory integration. In essence, interoceptive signals inform the organism about external objects just as exteroceptive signals inform the organism about the body. Changes in perceiving the body and objects beyond the body emerge if interoception and exteroception provide moderately deviating information about the same multimodal event.

This idea is not new at all and is, in fact, widely accepted in research on multisensory perception. Consider, for example, the well-known interactions between visual and haptic signals in manual grasping (Ernst & Banks, 2002; Helbig & Ernst, 2007). Here, the felt width of a hand opening provides information about the size of a grasped object just as the seen size of the object provides information about the width of the hand opening. If the objective sizes presented to vision and haptics are no longer identical (e.g., the perceiver is visually presented an object of 45 mm, but the felt-hand opening is 50 mm), the felt size of the hand opening and the visual size of the object change in a well-studied manner (described in more detail below). The same regularities hold true for other modality combinations, such as for the audiovisual domain (Alais & Burr, 2004).

The novelty of the present approach is thus not the discovery of multisensory integration but, rather, its application to a wide range of putative embodiment phenomena that are usually explained in phenomenon-specific ways. This application allows us to describe changes in object and body perception under manifold conditions by a common mechanism resting on only a few basic principles.

Given the huge number of studies of embodied perception published in the last decades, it is hardly possible to discuss all phenomena, their individual peculiarities, and specific explanations in detail within the scope of a single article. Moreover, we focus here on self-reported changes in perception typically assessed through judgment biases and refrain from considering other perception-related measures (e.g., reaction times to stimulation). As a result, several phenomena that might fall in the category of “embodied perception” are not included. For example, visual processing of objects near the hand are detected faster than objects further away (for a review, see Brockmole et al., 2013). The visual sensitivity of perceived body motions is enhanced for familiar compared with unfamiliar actions (for a review, see Blake & Shiffrar, 2007). Certain visual-object characteristics prime certain actions afforded by these characteristics such that manual responses are faster when the orientation of a handle of a presented object is congruent with the side of the manual response (e.g., Tucker & Ellis, 1998). Although we can think of expansions of our approach to capture such cases as well, we focus here on measures of what is perceived rather than how quickly the input is processed. In addition, we refrained from discussing potentially interesting links to related theoretical traditions, such as to the Gibsonian ecological approach that ascribes possibilities to act a fundamental role for perceptual experience (for a discussion of the relation between the ecological and recent embodiment approaches, see Witt & Riley, 2014).

We present our case in the following way. First, we discuss the core principles of how signals from different perceptual modalities are integrated and how this leads to perceptual biases in perception. Second, we illustrate how these principles apply to perceptual changes of the body and body-external objects in sensorimotor interactions. Third, we look closely at several embodied-perception phenomena and suggest that they can be explained by the principles of multisensory integration. Fourth, we revisit previous models that explain these phenomena in other ways and question the need for concepts beyond multisensory integration to explain them. Fifth, we discuss new questions that come with our framework.

## Principles of Sensory Integration and Perceptual Biases

In many interactions with the environment, different senses provide people with information about the same object. These mostly redundant pieces of information, often labeled as “cues,” are combined in perception taking the precision (i.e., reliability) of each signal into account to reduce overall signal variance when it comes to estimating an environmental property about which these various signals inform (“reliability weighting principle”; e.g., Ernst & Bülthoff, 2004; Welch & Warren, 1980). The more reliable a signal is, the more heavily it is weighted.

Second, the magnitude of multisensory integration increases the more signals appear to belong to the same event (“unity assumption”; e.g., Chen & Spence, 2017; Shams & Beierholm, 2010). Accordingly, the magnitude of multisensory integration can vary from a complete fusion of individual signals into a single percept, to partial integration, and a complete independence depending on causal inferences of the perceiver. Whether and how strong signals belong to the same event depends, on the one hand, on a variety of factors characterizing the given experimental situation, such as spatiotemporal contiguity, and covariation of the signals. On the other hand, it also depends on prior expectations about the causal relation between the signals and is thus a matter of experience (signals that often went together in the past more likely relate to the same external event) and of instructions. This principle thus suggests that the observer estimates the extent to which signals belong together according to their co-occurrence, commonality, correspondence, or correlation and that this determines the magnitude of integration.

**Fig. 1.**
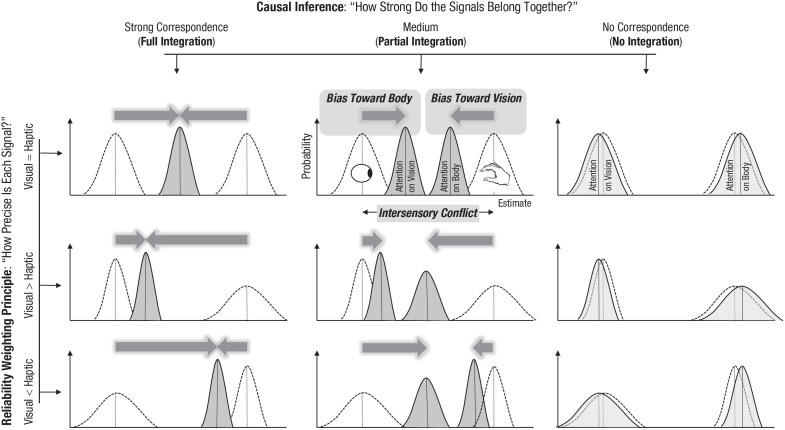
Changes in body and object perception in the presence of an intersensory discrepancy (here between a visually and haptically sensed size of an object as indicated by the eye and hand symbols) depending on the relative signal precision and causal-inference processes. Shown are fictitious likelihood functions reflecting the probability distribution of size judgments under unisensory (unfilled functions) and multisensory (filled functions) conditions (i.e., the *x*-axis represents different object sizes perceived under visual, haptic, or multimodal conditions, and the *y*-axis reflects how likely these perceptions are). Thus, the peaks indicate the most likely size for a given stimulus condition, whereas the width of such a function indicates how precise or reliable the underlying signal is (the wider it is, the less precise it is). Multisensory integration of visual and haptic signals results in a systematic change in visual-object perception in the direction of the haptic signal (“bias toward body”) and a bias in haptic perception toward the visual-object characteristic (“bias toward vision”). The magnitude of these mutual perceptual biases depends on two factors. The first factor is how precise is one signal compared with the other: The more precise signal attracts the less precise signal stronger than vice versa. The second factor is the observer’s belief of how strong the signals belong together: The stronger the supposed signal relation is, the stronger integration is and thus, the mutual attraction. In the case of full integration (left), the visual and the haptic signals are fused into a single percept. Accordingly, the mutual perceptual attraction covers the whole discrepancy between the visual and the haptic signals (i.e., the sum of both biases is equal to the discrepancy between visual and haptic signals). In the case of partial integration (middle), in contrast, there are separate percepts for visual (“attention on vision”) and for haptic (“attention on body”) object’s characteristics that do not coincide. If there is no integration, there are no such mutual-attraction biases (right).

Figure 1 outlines how these two principles entail changes in the perception of an observer’s body, which can be assessed by judgments of interoceptive signals, and of external objects, which can be assessed by judgments of exteroceptive signals, when there is a discrepancy between interoceptive and exteroceptive information. When looking at a handheld object, such as an apple, both visual and haptic signals inform people about the size of this object. Now consider that the visually sensed size of the object does not correspond to its haptic size (the visual size is, e.g., 45 mm, whereas the haptic size amounts to 50 mm). How large will the object appear in this situation?

In the case of full integration (also called “fusion”) that implies a strong signal correlation, the multimodal percept is a weighted average of visual and haptic estimates (Fig. 1, left). If the observer trusts visual and haptic signals to the same degree (i.e., if the reliability of haptic and visual signals is equal), the perceived size of the object will be exactly in between of what is visually and haptically sensed (e.g., 45 × 0.5 + 50 × 0.5 = 47.5 mm;^2^ see Fig. 1, left, upper graph). This means that the haptic perception of object size will be biased toward the visual size of the object (“bias toward vision”) to the same extent (e.g., 2.5 mm) as the visual perception of the object will be biased by the haptic size (“bias toward body”). If one of the signals is more reliable than the other, a stronger bias toward this signal should be observed (e.g., 45 × 0.8 + 50 × 0.2 = 46 or 45 × 0.2 + 50 × 0.8 = 49 mm; middle and lower graphs in the left column of Fig. 1). A full integration of discrepant visual and haptic signals thus entails changes in visual and haptic perception (e.g., of 2.5 mm each) that cover the whole magnitude of the intersensory discrepancy (i.e., 50 - 45 = 5 mm) and indicate a mutual attraction between these signals in perception depending on their relative reliability.

Signals are not completely fused into a single percept, but only partially integrated if their correlation is not very strong. In this case, the sum of the mutual biases does not cover the whole magnitude of the intersensory discrepancy (i.e., the sum of individual weights is less than 1). This implies that a judgment of visual-object size will not fully correspond to a haptic judgment of its size (Fig. 1, middle). Nevertheless, the reliability rule should still hold for this kind of integration. That is, the more reliable signal should affect the estimate of the other signal stronger than vice versa (see Fig. 1, middle, middle and lower graphs). In other words, a partial integration of discrepant visual and haptic signals also entails that the haptic perception of object size will be biased toward the visual size of the object (bias toward vision) and that the visual perception of the object will be biased by the haptic size (bias toward body). This mutual-perceptual attraction also depends on relative signal reliability. It is, however, smaller compared with full integration, and its magnitude varies as a function of the perceived signal relation (the stronger the perceived signal relation is, the larger the magnitude of integration is and thus of mutual attraction).

Integration makes sense only if there is a certain amount of correspondence or correlation between the signals. If this is not the case, the multimodal signals are not expected to be integrated and thus to attract each other (Fig. 1, right).

The reliability-weighting principle and the unity assumption have been formalized and thus allow inferring and testing of not only qualitative but also quantitative predictions (e.g., Ernst, 2006; Ernst & Bülthoff, 2004; Roach et al., 2006; Shams & Beierholm, 2010; for a tutorial on cue integration, see also Rohde et al., 2016). Moreover, they gained support in several domains of multisensory research and suggest that changes in body and object perception can originate from integration of interoceptive and exteroceptive signals related to a common event (or object) in the presence of multimodal discrepancies (see also Welch & Warren, 1980).

We now turn to instances of embodied perception. Typical studies on embodied perception resemble to some extent traditional multisensory research. Participants are usually asked to estimate some characteristics of external objects or of their own body (or its movements) while the relation between interceptive and exteroceptive information is systematically varied (i.e., a kind of multimodal discrepancy is introduced). Accordingly, the reported perceptual changes can arise, in theory, from signal integration following the outlined principles. However, unlike traditional multisensory studies (e.g., of manual grasping), interoceptive and exteroceptive signals often relate to physically distinct events (or objects) in studies on embodied perception. This might cast doubt on whether a conceptual link between traditional multisensory research and studies on embodied perception is justified. In the next section, we show that this link is in fact quite tenable by presenting some recent research that shows the basic principles of multisensory integration hold for scenarios in which interoceptive and exteroceptive signals relate to different events. This research also implies that the principle of unity (i.e., the unity assumption of multisensory integration) explained above is also valid when the multisensory signals have different origins.

## Integration of Interoceptive and Exteroceptive Signals Relating to Different Events: Bodily Interactions With Distant Objects

**Fig. 2.**
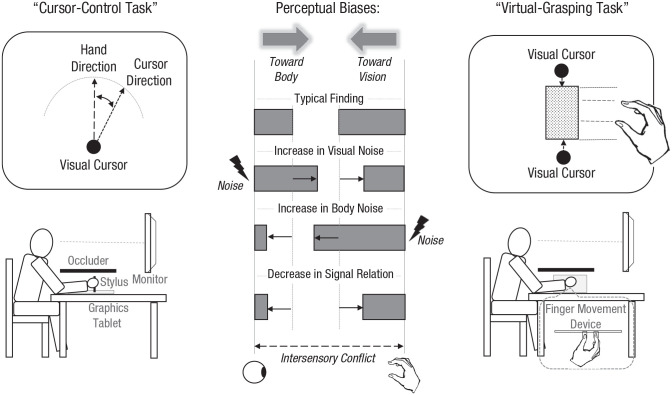
Experimental setups (left and right) and the main results (middle) of the cursor-control and the virtual-grasping tasks. In the cursor-control task, participants move a stylus on a graphics tablet in a certain direction. This movement is accompanied by a movement of a visual cursor. Then, participants judge the final position of the cursor or of the stylus. In this task, a conflict between hand- and cursor-movement directions is exploited to assess their mutual impacts. In the virtual-grasping task, participants enclose a visual object by two cursors (i.e., they place the cursors at two opposing edges of the object) controlled by their finger movements. A conflict is introduced between the distance between the fingers (i.e., hand opening during correct grasping) and the size of the object being enclosed, and the mutual influence of both is examined.

In one extensively used paradigm, called a “cursor-control task,” participants perform hand movements on a horizontal plane that control a visual cursor displayed in the fronto-parallel plane (Fig. 2, left) while the operating hand is occluded and can thus be sensed only proprioceptively. Following movement execution, participants judge the final positions of the cursor and of the hand, which are indicative of the perceived motion directions. These positions (and preceding motion directions) deviate from each other because of experimentally induced visuomotor rotations (e.g., a 45° rightward hand movement produces a 50° rightward movement of the cursor). The basic finding in this paradigm is that the visual movement direction of the cursor attracts the proprioceptively sensed direction of the hand movement, and vice versa, the proprioceptively sensed direction of the hand movement attracts (albeit to a lesser extent) the visual direction of the cursor (see the graph in the middle column of Fig. 2). Note that the relative magnitude of both biases depends on the relative reliability of exteroceptive (i.e., visual) and interoceptive (i.e., proprioceptive) information in accordance with the reliability-weighting principle. Adding noise to the visual signal leads to an increase of the bias toward body (i.e., bias of the visual judgment of the cursor toward the proprioceptive state of the hand) and a decrease of the bias toward object (i.e., bias of the proprioceptive judgment toward the visual cursor) compared with adding noise to the interoceptive signal (Fig. 2, middle; Debats et al., 2017b; Debats & Heuer, 2018b). In addition, in accordance with the unity assumption, the overall magnitude of both biases (i.e., their sum) that reflects the overall strength of integration decreases when the (real or inferred) signal covariation decreases (Debats et al., 2017a; Debats & Heuer, 2018a, 2020a, 2020b) or when hand and cursor move in an obviously incompatible manner (Liesner et al., 2020).

Very similar results are observed in a virtual-grasping task, in which participants enclose visual objects displayed in the fronto-parallel plane by manually controlled visual cursors and the perceived visual size of the object and the felt-finger posture (i.e., hand opening) are measured (Fig. 2, right). The size of the visual object usually attracts the judgments of the felt-hand opening, and vice versa, the felt-hand opening attracts (to a lesser extent) the visual judgments of object’s size when a visual-proprioceptive discrepancy is introduced (e.g., Kirsch et al., 2017). Moreover, in accordance with the reliability-weighting principle, the former bias decreases and the latter increases when the reliability of the visual information decreases (Kirsch & Kunde, 2019a, 2019b). In addition, in accordance with the unity assumption, integration strength decreases when the perceived signal relation decreases (Kirsch, 2021).

In the cursor-control task, there is a systematic covariation between kinematic variables of the hand and visual cursor movements (e.g., velocities). This likely promotes the observers’ inference that different objects (i.e., hand and cursor) are causally linked to each other and thus gives rise to sensory integration (Debats et al., 2017a). The situation is more complex in the virtual-grasping task, in which the relation between object size and finger movements is less obvious. Here, the visual cursors that are controlled by finger movements (i.e., the “grasping” aspect of the task) likely provide the critical evidence for the observer that hand opening and object size share common characteristics.

The results from these two paradigms show that sensory integration is a very flexible process that can comprise correlated signals related to spatially separated objects or events. Together with other findings suggesting that even initially unrelated arbitrary signals can be integrated (Ernst, 2007; Kaliuzhna et al., 2015), these results indicate that any interoceptive signals are integrated with any signals from other modalities provided a systematic relation between them is retained. This implies changes in the perception of the own (felt) body and of external (seen) objects in a wide range of environmental interactions.

In the next section, we consider several examples of such perceptual biases that are often described by specific mechanisms in embodiment-like terms. We show that these phenomena can be understood as “biases toward exteroception (e.g., vision of an object)” and “biases toward interoception (e.g., proprioceptively sensed body),” that is, as outcomes of integration of correlated interoceptive and exteroceptive signals in the presence of an intersensory conflict.

## Examples of Putative Embodiment in the Perception of Body and of External Objects

A common characteristic of many studies in the field of embodied perception is to induce a spatial or temporal discrepancy between what is interoceptively felt and what is sensed by another modality, such as vision, while exploring the effect of this manipulation on the perception of either body or external objects or both. Thus, the basic precondition for the application of sensory-integration principles, and thus for a potential influence on corresponding results, is met. Moreover, the general pattern of results is often the same across diverse paradigms and is in line with what has been observed in the cursor-control and virtual-grasping tasks. In particular, body perception (e.g., proprioception) is attracted by an object sensed through another modality (especially vision), whereas an exteroceptive object’s perception is attracted by interoceptive signals. We thus assume that these perceptual biases (i.e., the mutual attraction between interception and exteroception) are indications of multisensory integration. In what follows, we explicate this claim for several groups of phenomena reported previously.

### Rubber-hand illusion

One prominent correlate of the rubber-hand illusion is the so-called proprioceptive drift, a shift of the perceived location of the real hand toward the fake hand. This shift represents an attraction of the visually occluded, interoceptively sensed, own hand by a spatially displaced and visually sensed fake hand and can thus be considered as a bias toward vision. This bias is reduced or completely disappears when own and rubber hand are stroked asynchronously (e.g., Botvinick & Cohen, 1998), at different parts of the hands (e.g., Riemer et al., 2014), in anatomically dissimilar ways (e.g., Tsakiris & Haggard, 2005) or when the spatial distance between them increases (e.g., Kalckert & Ehrsson, 2014; for reviews, see also Braun et al., 2018; Kilteni et al., 2015; Riemer et al., 2019; Tsakiris, 2010). These findings are fully consistent with the unity assumption of sensory integration and suggest that when the perceived relation between the real hand and its visual counterpart decreases (i.e., object unity is violated), the illusion decreases (for related assumptions, see also e.g., Armel & Ramachandran, 2003; Kilteni et al., 2015). Moreover, some recent studies on this illusion have revealed a perceptual bias of the opposite direction (i.e., bias toward body)—a shift of the perceived location of the fake hand toward the real hand (Erro et al., 2018; Fuchs et al., 2016). This finding further supports the multisensory origin of the illusion indicating a simultaneous mutual attraction between interoceptive and exteroceptive signals. Thus, the rubber-hand illusion is a robust observation that can be explained by multisensory integration alone (i.e., disregarding any embodiment-like terms such as “body ownership”).

This illusion and its numerous variants (e.g., Ehrsson, 2007; Lenggenhager et al., 2007) have several characteristics in common with the effects of wearing prism glasses (e.g., Hay et al., 1965), of visuomotor adaptation to misaligned visual feedback (e.g., Salomonczyk et al., 2012), and with cursor-control tasks extensively studied by Debats and colleagues. In essence, in all of these paradigms, researchers measured the proprioceptive perception of a body part and/or the perception of an (usually visual) object that supposedly represents that body part in the presence of a spatial discrepancy between both. Accordingly, interoceptive and exteroceptive signals are interrelated to a certain degree, and the observed attractive biases in body and object perception can well be considered as resulting from multisensory integration in the presence of an intersensory conflict.

### Perceived body size and visual-object perception

Sensory integration does not seem to be restricted to the rubber-hand illusion and related phenomena in which an external object, such as a cursor or an artificial hand, represents a body part of the observer. The perception of objects that are clearly distinguishable from the body and its movement can also be altered in accordance with multisensory-integration principles, as suggested by the results from the virtual-grasping task described above. Some interesting putative embodiment phenomena fall in this category. In particular, the perceived size and distance of visual objects are influenced by an experimental variation of the perceived size of the body.

For example, Linkenauger and colleagues (2015) observed that a virtually extended arm produced a decrease in the perceived distance to a target object after reaching experience compared with a small virtual arm. In a similar vein, an object appears to shrink (expand) when the size of the hand and the object are virtually enlarged (demagnified; Linkenauger et al., 2010). How can these findings be related to the present sensory-integration approach? Consider that reaching an object using a large virtual arm entails a discrepancy between the interoceptively sensed and the visual arm positions corresponding to the object’s location. For a given actual object distance, the felt position of the arm signals a smaller object distance for a long virtual arm than for a small virtual arm (because the hand is closer to the body during reaching the object in the former than in the latter condition). Thus, a multisensory percept of object distance should be more strongly biased toward the body with a large virtual arm compared with a small virtual arm. In a similar vein, grasping an object via a virtually distorted hand entails a conflict between felt- and seen-hand representations. In the virtually larger (smaller) hand condition, the felt-hand aperture (e.g., during potential object grasping) is smaller (larger) than its visible counterpart. If interoceptive signals of the hand are taken into account in the estimation of object’s size (as also generally agreed by Linkenauger et al., 2010), then the observed pattern (i.e., a decrease in perceived object size with an increase in virtual size of the hand) should emerge following integration of interoceptive hand and visual-object information. In other words, the results of these studies can be considered as biases toward body by analogy to the virtual-grasping task that we used.^3^

This conclusion is further supported by two independent yet complementary findings. First, when observers use a tool, such as a stick or a rake, for reaching distant objects, the arm feels elongated (Cardinali et al., 2009; Sposito et al., 2012). Second, in comparable experimental situations, objects reachable by the tool appear closer to the body (Davoli et al., 2012; Witt, 2011b; Witt et al., 2005; Witt & Proffitt, 2008; for the same effect after merely observing an actor, see also Bloesch et al., 2012). The perceived location of an external object is thus attracted by the felt-hand location, whereas the perceived location of the hand is attracted by the location of the object. In other words, a bias toward body is accompanied by a bias toward vision, in line with the proposed integration account. Here, the effective part of the tool (e.g., the tip of a stick) resembles a cursor controlled by the hand (as in the cursor-control task), that is, a kind of a visual counterpart of the felt-hand location. Accordingly, the felt extension of the arm following tool use is basically a perceptual bias toward this visual counterpart of the felt-hand location (i.e., bias toward vision analogous to proprioceptive drift and related distortions). The inverse bias (i.e., decrease of perceived egocentric distance to the object) likely arises because the location of the effective part of the tool coincides with the location of the object during reaching that object. This likely provides evidence that the hand and the visual object belong together (or are correlated) and leads to a multimodal percept of the object location. The hand location sensed by interoception is an integral part of this percept.

### Walking, throwing, and jumping

The spatial correspondence between an observer’s body and external objects, which informs the observer that both are related (and correlated) and thus promotes an integration of interoceptive and exteroceptive signals, is rather obvious for the phenomena mentioned so far. Such correspondence applies, although in a less obvious manner, to many daily interactions with the environment as well. As an example, consider walking from one place to another. Introducing a discrepancy between visual and interoceptive cues of distance during walking leads to systematic biases in the perception and subsequent walking. Specifically, accelerating optic flow relative to the corresponding biomechanical rate of walking decreases the subsequent distance walked to a target object, and vice versa, decelerating optic flow increases the subsequent walking distance (Durgin et al., 2005; Mohler et al., 2007; Rieser et al., 1995; Thompson et al., 2005). Moreover, in the latter condition, the perceived target distance increases (compared with a no-conflict condition; Proffitt et al., 2003). The former biases (in walking distance) reflect changes in the perception of interoceptive signals related to walking a certain distance toward the visual signals relating to that distance and can thus stand for a bias toward vision. The latter bias (in perception of target distance) is the opposite bias toward body. These conclusions are further supported by the results of Campos and colleagues (2012), who measured the perception of distances just walked. The authors observed that the perceived distances were in between of what visual and interoceptive cues suggested, in accordance with the reliability-weighting principle of sensory integration (for reviews on sensory integration during self-motion, see also Campos & Bülthoff, 2012; Fetsch et al., 2010). Thus, perceptual biases observed in the research on walking can be conceptualized in the same way as biases observed with artificial body limbs, tools, and other objects, whose characteristics are correlated with the interoceptive body signals while a discrepancy between interoception and exteroception is experimentally introduced.

There are several similar observations in which a relation (or correlation) between what is bodily felt and what is sensed by another modality is not very obvious at first glance while a conflict between both types of signals is present. Hills are judged as steeper when wearing a heavy backpack compared with not wearing a backpack (Bhalla & Proffitt, 1999; see also Schnall et al., 2010), distances are judged as larger when throwing a heavy rather than light object (Witt et al., 2004), and walls are judged to be smaller by parkour experts than by novices (Taylor et al., 2010), to name a few examples (for reviews, see also e.g., Proffitt & Linkenauger, 2013; Witt, 2011a). Despite a partly sharp criticism of some of these and related findings (we return to this issue later), they are plausible when considered from the multisensory perspective. The one additional assumption necessary is that planning or simulating a certain motor activity comes with memory retrieval of interoceptive signals associated with that motor activity, an assumption that has received a lot of support (e.g., Brown et al., 2013; Pfister, 2019). Here, experimental manipulations change the relation between these retrieved interoceptive signals and visual signals related to that motor activity. This corresponds to an introduction of intersensory conflict, and the measured effects might indicate biases toward body. To be more precise, consider that climbing a steeper hill, covering a larger spatial extent, or jumping a higher wall is usually associated with an increase in action difficulty or demand. Accordingly, making a potential movement to a target object more difficult, by a backpack, heavy ball, reduced fitness, or action ability, corresponds to climbing a steeper hill, covering a larger spatial extent, or jumping a higher wall under less demanding conditions. If variables related to a potential action (e.g., perceived effort; see e.g., Proffitt, 2006) enter sensory integration in the perception of external objects, then an increase of the perceived slant, spatial extent, or height should occur because the interoceptive cues associated with these actions would signal a steeper slant, larger extent, or a larger height.

### Body orientation

The effects of body orientation on the perceived distance and orientation of objects are other instances for putative embodiment phenomena that can be explained in terms of the present multisensory approach. Harris and Mander (2014) observed that distances are underestimated when participants are lying supine (or feel as if they were lying supine) than when the body is in a usual upright orientation. Changing body orientation certainly causes a type of cross-modal conflict (i.e., a change in the usual relation between interoceptive and exteroceptive signals). Accordingly, the observed effect can be considered as a bias toward body if the body signals a smaller distance when oriented supine (cf. also Harris & Mander, 2014, p. 2). Alternatively, this bias can also be an outcome of a reweighting of visual and interoceptive signals (i.e., weighting vision more heavily than interoception) during sensory integration given that the perception of body orientation when lying supine is rather poor (Harris & Mander, 2014, p. 6; cf. also McManus & Harris, 2021). The impact of body orientation is not limited to distance perception. When the orientation of a visual and a haptic stimulus is judged relative to gravity and the body is tilted, the visual estimate is biased toward the current body orientation, whereas the haptic estimate is biased away from the body orientation (e.g., Fraser et al., 2015; for a review of related effects, see also Harris et al., 2015). The former bias might represent the bias toward body (i.e., a perceptual attraction of the visual stimulus by the current body orientation). The latter effect, in contrast, could reflect a bias in the perception of the current body orientation toward the gravity vertical^4^ (i.e., a kind of bias toward vision; cf. e.g., Nestmann et al., 2020). Despite these rather specific conjectures, there is increasing agreement that biases in the perception of verticality arise from integration of different sensory cues in accordance with the reliability-weighting principle and the unity assumption (e.g., de Winkel et al., 2018; Mittelstaedt, 1983; Nestmann et al., 2020).

### Temporal binding

A mutual perceptual attraction between interoception and exteroception that, we argue, is indicative of sensory integration is evident beyond the visual modality as well. One prominent example is the temporal attraction between an action and its auditory effect called “temporal binding” (Haggard et al., 2002). The crucial empirical observation is that the perceived time of a key press is shifted toward the time of a sound (action binding), and vice versa, the perceived time of the sound is shifted toward the time of the key press (effect binding) when the key press is followed by the sound. The key press and the sound are causally related and overlap in time while a temporal discrepancy between them remains because the sound follows the key press with a certain delay. Accordingly, the mutual attraction between these events corresponds to spatial distortions between causally related proprioceptive and visual events in the spatial domain (i.e., in the cursor-control tasks, rubber-hand illusion, etc.; see also e.g., Kawabe et al., 2013; Legaspi & Toyoizumi, 2019; Moore & Fletcher, 2012). In fact, the perceived temporal attraction between the tactile event (key press) and auditory event (tone) is subject to exactly the same factors as the perceived spatial attraction between proprioceptive events (hand movement) and visual events (cursor movement). Specifically, the magnitude of both biases depends on the relative reliability of information related to the action and its effect (Cao et al., 2020; Klaffehn et al., 2021; Wolpe et al., 2013; Yamamoto, 2020) rather than on the presence of action intention (Buehner, 2012; Buehner & Humphreys, 2009; Kirsch et al., 2019; Ruess et al., 2020; Suzuki et al., 2019). Thus, action-binding and effect-binding phenomena correspond to biases toward exteroception (“bias toward audition,” i.e., an analog of bias toward vision) and interoception (bias toward body), respectively, that emerge when integrating correlated and discrepant multimodal signals.

### Repulsion phenomena

The phenomena outlined so far indicate a perceptual assimilation or attraction between interoceptive and exteroceptive signals. However, intersensory discrepancies not only entail a mutual perceptual attraction between interoception and exteroception but can also lead to repulsion phenomena. From a multisensory perspective, these effects indicate that the signals are not considered to belong together or that the conflict between them is too large (e.g., Antusch et al., 2021; Körding et al., 2007). Consequently, they are not integrated and, rather, are pushed away from each other (although it is not well understood why the latter happens).

Although such effects are usually not discussed in the embodied-perception literature, they might underlie some prominent putative embodied phenomena (van der Hoort & Ehrsson, 2014, 2016; van der Hoort et al., 2011). In the original paradigm, a “full body illusion” is induced by synchronous touching of observers’ body and of an artificial body shown via a head-mounted display. This changes the perception of the own and the artificial body analogous to the rubber-hand illusion. That is, participants’ reports indicate that they perceive their body as smaller/larger when the artificial body is small/large. This result is consistent with a mutual attraction of interoceptive and exteroceptive signals (i.e., with their integration) in line with several phenomena described above. More importantly here, when asked to judge the size of or egocentric distance to a distant object (cube), repulsion rather than attraction phenomena emerged. An increase in felt body size decreased the estimated size and distance of the object, and vice versa, a decrease in felt body size increased the estimated size and distance of the object. From the perspective of the present approach, this pattern indicates that interoceptive signals and object information were treated as unrelated and were thus not integrated but, rather, were kept separate. This possibility appears plausible because no (real or imagined) actions that could impose a possible interrelation between the body and the object (like, e.g., in the virtual-grasping task) were required or promoted by the experimental situation.

### Sensory integration and action planning

Some effects of interoceptive changes in the perception of external objects arise early, during action planning (i.e., before critical interoceptive input is available; e.g., Kirsch & Kunde, 2013). The so-called ideomotor approach of action control that received a wide support in the last decades suggests that planning a goal-directed body movement involves, or actually is brought about by, the activation of interoceptive and exteroceptive effects of that movement (e.g., Elsner & Hommel, 2001; Kunde, 2001; for reviews, see e.g., Pfister, 2019; Shin et al., 2010). In other words, actions are assumed to be initiated and controlled by anticipation of their sensory consequences. According to this approach, reafferent body-related input is anticipated during action planning and thereby enters multisensory integration before actual reafferent stimulation being actually present.

Keeping anticipated stimulation during action planning in mind, we revisit the famous backpack example discussed before in a bit more detail. Our reasoning here is very similar to the original explanation of this and similar effects. It has been proposed that the visual perception of a hill “is influenced by people’s physiological potential to climb it” (see e.g., Bhalla & Proffitt, 1999, p. 1093). This implies that observers plan or at least anticipate (or simulate) ascending the hill while judging its incline (even though they did not translate this plan into action in the original studies; see also Proffitt et al., 2003; Witt & Proffitt, 2008). Planning to climb a hill (like any other intentional motor activity), we argue, goes along with anticipation of the sensory consequences of that action, including interoceptive changes that correspond to climbing the hill, such as changes in the anticipated leg-muscle tensions when working against gravity. Now consider that the observer knows from experience that the steeper the hill is and the more encumbered she is by the weight of her backpack, the more muscle tension is felt. Thus, when wearing a medium-heavy backpack, an incline of 20°, for example, is associated with less muscle tension than an incline of 30°. Likewise, an incline of 25°, for example, feels like 30° when wearing a very heavy backpack and like 20° when wearing a very light backpack. In other words, a certain muscular feeling signals a certain incline given the circumstances. This corresponds to the interoceptive estimate visualized in Figure 1 as a likelihood function marked by the hand symbol. Critically, this estimate varies as a function of the backpack weight being 30° and 20° for a heavy and light backpack conditions. In the example above, the visually sensed incline (i.e., the visual estimate visualized in Fig. 1 as a likelihood function marked by an eye symbol) is 25°. Assuming that the interoceptive and exteroceptive estimates are equally reliable and are fully integrated, the multimodal estimate of the incline would amount to 27.5° and 22.5° for the heavy and light backpack conditions. Accordingly, the hill is judged as steeper when wearing a heavy backpack, basically because what is sensed by the eyes is attracted by what the body *would feel* when climbing the hill (hence, bias toward body). In sum, when asked to judge the steepness of a hill, the anticipated interoceptive cues enter the judgment of the slope, as do actual visual steepness cues. Consequently, anticipated interoceptive cues and visual cues mutually affect each other and thus determine changes in the multimodal slope perception.

## Implications

### Sensory integration as a common explanation for various embodiment phenomena

We raised several empirical observations and outlined how they can be related to basic principles of sensory integration. In essence, these observations can be understood as changes in the perception of either observer’s body (i.e., as biases toward vision) or of external objects (i.e., as biases toward body) that arise when the perceptual system integrates related (and correlated) yet deviating signals from different modalities. Thus, many putative embodiment phenomena, we argue, are indications of sensory-integration processes that follow well-known principles, such as the reliability-weighting principle and the unity assumption.

To avoid misunderstandings, some important notes are in order here. We not only state that multisensory integration in general and its certain principles in particular are somehow related to the manifold changes in body and object perception listed in the previous sections. We in fact suggest that these concepts are sufficient to explain the mentioned putative embodiment phenomena. One can thus consider these concepts as descriptions of when, how, and to what extent interoceptive signals shape perceptual processes (i.e., of the core mechanism of “embodied” perception).

We are also aware that the reliability-weighting principle and causal-inference processes (i.e., unity assumption) have been already acknowledged in some of the outlined paradigms. However, most of the findings are still often explained in embodiment-like terms that are not consistent with these principles. In the next sections, we touch on these previous accounts, point to their limitations, and outline some theoretical implications of our approach.

### Previous accounts and their limitations

A common peculiarity of previous embodiment approaches to the manifold effects in the perception of body and of external objects refers to the fact that interoception is considered as more important or more fundamental than exteroception.^5^ Specifically, the overemphasis on the role of the body, or as we explained, interoception that informs about the body, is just one side of the coin when looking at such phenomena from the perspective of multisensory integration. Looking at all mutual influences of interoception and exteroception will provide a more comprehensive picture of these phenomena.

#### Is the rubber-hand illusion an indicator of “body ownership”?

The proprioceptive drift of the own hand toward a rubber hand is commonly considered as one of the indicators of body ownership (Butler et al., 2017; Kilteni et al., 2015; Riemer et al., 2019; Tsakiris, 2010). That is, an external object is assumed to become an integral part of the own body after a certain spatiotemporal correspondence between body and that object is experimentally established. In other words, the internal representation of an external object (i.e., of an artificial hand) is literally “devoured” by the representation of the real hand. We suggest instead that proprioceptive drift is not more than it is (i.e., a measure of interoceptively sensed hand position). The phenomenon as such is not in question, but it can be explained by multisensory integration alone. No theoretical overhead in terms of “ownership” is needed. A similar reasoning can be applied to tool-use studies. Here, stating that a tool became a part of the body because of a felt elongation of the joint is a similar explanatory overhead (e.g., Cardinali et al., 2009).

To better understand our argument here, consider that the rubber-hand illusion belongs, in terms of traditional multisensory research, to “visual capture” phenomena in which visual rather than interoceptive signals dominate. A related phenomenon, is, for example, that the hand feels displaced in the direction of where it looks to be when wearing prism glasses^6^ (Hay et al., 1965). The common body-ownership description, however, emphasizes the opposite (i.e., a kind of interoceptive “capture” or “spread”) while the visual signal (i.e., the main driving force behind the bias from a multisensory point of view) is basically neglected. From the perspective of multisensory integration, it is equally correct to say that a seen rubber hand is “embodied” as it is to say that an own felt hand is “environmentificated.” The label “embodiment,” we feel, overemphasizes just one aspect for no good reasons.

#### Do people scale sensory input in motor units?

Several effects of body-related (i.e., interoceptive) variables on (mostly visual) perception of body-external objects are often ascribed to a kind of scaling of exteroceptively sensed information in interoceptive units. Here, the body and its potential to move is considered as a reference or ruler for the perception of environmental objects (Proffitt & Linkenauger, 2013; see also e.g., Harris et al., 2015; van der Hoort & Ehrsson, 2014; Witt, 2011a). For example, the slant of a hill is perceived in terms of the estimated likelihood an observer’s body was able to climb that hill (the lower the likelihood, the steeper the hill).

What researchers in this research field usually do is to manipulate a certain interoceptive variable while keeping visual input constant and then test whether this manipulation affects the visual perception of a certain stimulus characteristic. Finding such an effect is considered as indication for the proposed scaling process. There is, however, nothing in the used designs or in the empirical data as far as we can see that necessitates the claim that interoception provides the units in which exteroceptive events are measured.

The proposed scaling mechanism has already been criticized on various grounds (e.g., Firestone, 2013; Firestone & Scholl, 2016), and the proponents of this mechanism responded to the raised criticism by providing counterarguments and by defending their original claims (Clore & Proffitt, 2016; Proffitt, 2013; Witt, 2015; Witt et al., 2016). One argument in favor of body scaling in this debate seems to be theoretical rather than empirical in nature (empirical issues are raised later). In spatial perception, sensory information must be transformed into “semantics of human experience,” and the own body, or more precisely, interoception informing about the body, seems situated to provide such semantic units (see e.g., Proffitt, 2013, p. 474). The core of this assumption is not new (see Note 5), and neither is its criticism: The representations of the body and its movements are not sufficiently accurate to serve as a reference in visual perception, and they have no more or less meaning as other mental events (Pillsbury, 1911, pp. 85, 98). Thus, the proposed scaling mechanism seems debatable for theoretical reasons, not only empirical reasons.

#### Is temporal binding an indicator of the “sense of agency”?

Another example of an unnecessary theoretical overhead is the interpretation of temporal binding that is commonly considered as a proxy for the “sense of agency,” thus the term “intentional binding” (e.g., Braun et al., 2018; Haggard, 2017; Haggard & Tsakiris, 2009; Moore & Fletcher, 2012). This interpretation mainly rests on the reduction or absence of temporal binding with involuntary actions (e.g., Engbert et al., 2007, 2008; Haggard et al., 2002; Moore et al., 2009; Tsakiris & Haggard, 2003). This research, however, suffered from methodical shortcomings (e.g., Hughes et al., 2013; Kirsch et al., 2019),^7^ and more recent studies have demonstrated temporal binding in the absence of intended actions or effects that is comparable with the binding observed with intended actions and effects (Kirsch et al., 2019; Ruess et al., 2020; Suzuki et al., 2019; see also e.g., Buehner & Humphreys, 2009). Moreover, when an impact of action intention was found, it was rather small (see e.g., Borhani et al., 2017, Fig. 2).^8^ Note that studies that used transcranial magnetic stimulation reported a reversed binding effect (i.e., a repulsion between action and its effect; Haggard et al., 2002; Tsakiris & Haggard, 2003), which perfectly aligns with causal-inference processes as the likely source of the observed results but is hard to explain by action intention (Buehner, 2015; Desantis et al., 2011; Kirsch et al., 2019). The state of research can thus be summarized by saying that a role of action intention for temporal binding is mixed at best. In any case, there is no unequivocal evidence for the explanation of temporal binding in terms of the sense of agency.

This short overview of central claims of previous approaches and their empirical underpinnings shows that explanations going beyond multisensory integration are not well supported. When considered from the perspective of traditional multisensory research, all of these accounts focus on only one of two important aspects of the phenomena and overlook the other. In particular, all of them emphasize interoception and basically neglect exteroception. In the next section, we outline how the present approach can correct this unilateral view.

## Some Fundamentals of the Multisensory Approach to Embodiment Phenomena and Their Implications

The term “embodiment” has several connotations. Accordingly, some embodiment researchers would perhaps agree with the present multisensory approach and appreciate it for its clear and *formalizeable* description of the impact of interoception (i.e., of the core mechanism of embodiment). In other words, the multisensory integration could be construed as a mechanism through which something is “embodied.” On that take, “embodiment” means that interoception somehow affects perception. However, if one accepts the multisensory view, there would be not much left to be explained. Concepts like “embodiment,” “body ownership,” “sense of agency,” “intentional binding,” or “scaling in body units” lose their explanatory (and even descriptive) power and thus will be redundant or, at best, will serve as ambiguous heuristics. They still might be helpful to describe other phenomena. But these concepts are unnecessary to explain various empirical observations (e.g., proprioceptive drift, temporal binding, changes of space perception) that allegedly measure them.

After all, is there anything “special” about embodied perception? From the perspective of multisensory perception, the answer is “No.” These phenomena follow similar rules as multisensory integration in general. The only way to save a special status of embodied perception was to assign interoceptive signals a special role. There might be reasons to do so. For example, interoceptive signals are highly diagnostic regarding which part of the environment (the part we call “biological body”) should be safeguarded against physical threat (Liesner & Kunde, 2021). It is thus interesting that exteroceptive signals after being aligned with interoceptive signals in multisensory integration are subject to attempts to shield them against such threat (Armel & Ramachandran, 2003). In addition, proprioceptive signals, real and anticipated, play a key role in controlling body movements. Finding that proprioceptive signals are integrated with exteroceptive signals, as in temporal binding, is interesting because such proprioceptive signals are highly diagnostic for own body movements. Thus, although researchers may assign body-related signals a special role for certain reasons, the mechanics that drive the interaction between interoception and exteroception are not special at all.

What the present multisensory approach suggests is, in essence, that both interoception and exteroception are a priori equivalent. What counts is the relative signal precision and the perceived signal relation. Taking this into account, interactions between interoceptive and exteroceptive channels are governed by the same law as interactions among exteroceptive channels, such as vision and audition. Accordingly, interoceptive signals, we argue, shape multimodal perception just as any other modality does.

**Fig. 3.**
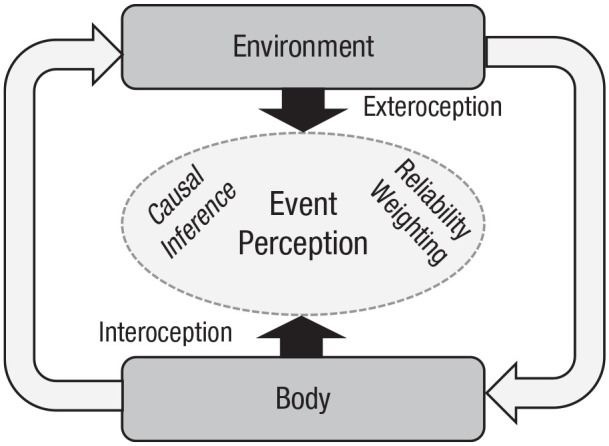
Multisensory event perception in the context of the interaction between body and environment.

This reasoning implies a dynamic symbiotic relation or unity between environment and body (or interoception and exteroception) in mind rather than primacy of interoception, as suggested by previous approaches.^9^ Changes in body and object perception can thus be understood as a result of an attempt to unify partly discrepant sensory information about related and correlated events to produce a kind of equilibrium state between inside and outside of the organism in the particular situation (see Fig. 3). The evolutionary function to do so is to achieve the most accurate (i.e., reliable) estimate of an internal or external event (see e.g., Ernst & Bülthoff, 2004). This view, we believe, captures the numerous intriguing findings accumulated in the last decades more appropriately than any previous explanations in embodiment-like terms.

How far a certain phenomenon is multisensory in nature (i.e., how strong integration is) and how strong the impact of interoception is (i.e., its relative weight) depends on the location of this phenomenon along two continuums. First is from a strong relation between interoception and exteroception to their independence (unity assumption). Second is from a strong dominance of interoception over exteroception to strong dominance of exteroception over interoception (reliability weighting). The multisensory approach allows researchers to describe (qualitatively and quantitatively) to what extent interoception affects perception under manifold conditions. For example, the rubber-hand illusion appears to be based on strong integration of interoception and exteroception (because signals are fused^10^), whereas temporal binding in agency research rather reflects weaker partial coupling (because body- and object-related judgments usually do not coincide). Likewise, the rubber-hand illusion reflects a dominance of exteroceptive over interoceptive signals (because the bias toward vision is stronger than the bias toward body), whereas the opposite is true for temporal binding (because the perceived temporal shift of an external action effect toward the action is usually larger than that the perceived shift of the action toward the external effect). We believe that many putative embodiment phenomena can be arranged in this way. However, to be able to do so, more empirical data are need.

## Future Directions

The here proposed transfer of findings from traditional multisensory research via cursor-control and virtual-grasping tasks to diverse other paradigms leaves gaps. We believe, however, that these gaps could be filled in an informed way in future studies with straightforward predictions. For example, degrading the visual signal of a fake body part in the rubber-hand paradigm should decrease the proprioceptive drift but increase the inverse bias (i.e., “visual drift”). In studies on virtual body alterations and object perception, body perception could also be measured. Such an estimate of a body part should be in between the real and virtual characteristics of that body part. In studies on body orientation, the perceived orientation of the body should be in between its real and usual upright orientation. In studies on “action-specific perception” (see especially the “Walking, Throwing, and Jumping” section), researchers could measure some interoceptive judgements associated to what is construed as “effort,” such as the weight of a backpack, and observe that they are attracted by critical visual characteristics of the environment (e.g., slope inclination, distance to be covered, or height of a wall). For example, when asked to climb a hill, observers might overestimate the felt weight on their back when facing a steep rather than shallow hill. Moreover, because the interoceptive cues can be assumed to be rather fuzzy, the judgments of the weight of a backpack should be more heavily influenced by the seen steepness of a hill than the judgments of the hill steepness are influenced by the weight of a backpack.

Here, a note is in order with respect to some findings from the field of action-specific perception that drew severe criticism previously (e.g., Durgin et al., 2009, 2012; Firestone, 2013; Firestone & Scholl, 2016). The raised arguments range from empirical aspects, such as replicability of the results and feasibility of the methods, to theoretical issues, such as implausibility of conclusions. We believe that the present approach can help to resolve this controversial debate, at least to some extent. Consider, for example, the fact that some influences of interoceptive variables on visual perception could not be replicated (de Grave et al., 2011; Molto, Morgado, et al., 2020). This could, of course, mean that the effect does not exist (although conceptually similar phenomena seem reliable; see e.g., Molto, Nalborczyk, et al., 2020). However, this could also reflect that biases toward body are often much smaller than the inverse effects. In other words, high reliability of visual signals might simply overshadow influences of other perceptual modalities. A simple test for this claim would be to degrade the precision of the critical visual signal and to repeat the study in question. Another prominent objection relates to task instructions. Wearing a heavy backpack no longer affected the judgments of hills (cf. Bhalla & Proffitt, 1999) when the participants received a cover story relating to the presence of the backpack (Durgin et al., 2009; for related findings, see also Durgin et al., 2012; Firestone & Scholl, 2014; Shaffer et al., 2013; Williams et al., 2012). This was taken as evidence that the backpack impact on visual perception actually reflects a change in participants’ judgments rather than in their perception. However, communicating to an observer that two objects that are potentially related under natural conditions (i.e., hill and backpack) have nothing in common certainly affects observer’s causal inference toward perceptual disintegration of these objects. In other words, the observed impact of the instruction does not necessarily inform about whether the effect is perceptual. It could merely reflect a weakening or breakdown of sensory integration that is part of natural perception. To avoid misunderstandings, we do not encourage disregarding the raised objections but advocate their critical reassessment in the context of the present framework.

Sensory signals not only attract but also sometimes repel each other in perception (see also “Repulsion Phenomena” section). Attraction is in accord with signal integration with intersensory discrepancy, and repulsion presumably indicates that the signals are kept separate. Thus, the basic idea here is that causal-inference processes determine whether perceptual attraction or repulsion will be observed (see also Fig. 1). Appearing simple at first glance, this issue is getting complex when findings across different paradigms are compared (see e.g., Zwickel & Prinz, 2012). In our earlier experiments, for example, we consistently observed an assimilation bias in that an estimate of visual-object distance was attracted by a planned hand-movement amplitude (Kirsch & Kunde, 2013). Following a very similar rationale in judgments of visual locations, however, we observed a contrast effect in that the planned movement direction repelled rather than attracted the visually perceived target location (Kirsch & Kunde, 2014). Which specific factors are responsible for such a change in the putative decision to integrate or not is unknown at present, as are the conditions for repulsion.

Some researchers suggested that if two to-be-distinguished events share a common feature,^11^ this feature is “occupied” by either one of the events and thus less available for the other event (Hommel, 2004; Hommel & Müsseler, 2006). Such a feature occupation can lead to repulsion effects (whereas feature activation is assumed to produce attractive biases). This explanation rests on the “theory of event coding,” which suggests that action and perception share common cognitive representations of events, so-called event files (e.g., Hommel, 2019; Hommel et al., 2001). We partly adopted the vocabulary of this approach and share some basic ideas, such as action production rests on codes of intended stimulation. Accordingly, there is some common theoretical ground of these research directions. It is, however, difficult to say how the principles of multisensory integration in general and the magnitude of a perceptual bias in particular can be specified in terms of the common coding approach that explicitly abstracts from different sensory modalities to describe interactions between perceptual- and action-related processes (e.g., Hommel et al., 2001, p. 862). Although we generally agree with cognitive representations of sensorimotor events in terms of their rather abstract features (e.g., “object size” or “hand opening”), we feel that the impact of modalities is underestimated in the common coding framework, even though this depends on how a “feature” is conceptualized. We thus feel that the model does not yet capture easily the sort of multisensory interactions discussed here, although it might be expanded in the future to do so.

## Summary

Various research has documented changes in the perception of external objects and observers’ bodies under diverse task conditions. Many of these effects have been ascribed to a primary role of the body and its action. Yet viewed from the perceptive of mechanisms that conceivably cause these effects, there is no reason to assign body-related signals a special role. The human body and its movements just provide sensory signals, as other objects do. Changes in the perception of objects and the actor’s/perceiver’s body arise from well-known principles of multisensory integration of correlated signals. This view implies a context-dependent mélange of interoceptive and exteroceptive signals in body and object perception being expressed in perceptual biases in the presence of intersensory discrepancies. The vast majority of observations are in line with this approach at present. More research is, however, needed to better evaluate its scope and limitations.

## Transparency

*Action Editor:* Laura A. King

*Editor:* Laura A. King

## Notes

1. The broader term “embodied cognition” encompasses more aspects as outlined by Wilson (2002): (a) Cognition is situated, (b) cognition is time-pressured, (c) people off-load cognitive work onto the environment, (d) the environment is part of the cognitive system, (e) cognition is for action, and (f) offline cognition is body based. Here we focus on influences of body states on perception.

2. The multiplication factors “0.5” are relative weights assigned to visual and haptic information and can vary between 0 and 1 and add up to 1 in case of full integration.

3. This interpretation implies that the hand and the object are experienced as related, that is, the observers are assumed to mentally apply some actions (e.g., reaching and grasping) to these objects through virtually magnified or minified limbs. This assumption is similar to the original interpretation of these effects suggesting that objects are perceived in terms of observers’ action capabilities. If the observers do not plan to interact with these objects, then the reported effects would speak for a repulsion of interoceptive and exteroceptive signals indicating that these signals are kept separate rather than for their integration. We return to this issue later.

4. If the body is tilted to the left by 30°, for example, and its perceived orientation is about 25°, then a haptic judgment of the gravity vertical should be biased by about 5° to the right. This should be so because after a (hypothetical) mental rotation of the body to the upright position, this position should be felt as tilted to the right by about 5°.

5. This emphasis of interoceptive (or motor) processes for perception can be viewed as a renaissance of so-called motor theories of perception that can be traced back to the works of prominent people such as George Berkeley, Hermann Lotze, and Hermann von Helmholtz (for historical reviews, see e.g., Briscoe & Grush, 2015; Scheerer, 1984). One of their core ideas is that the body constitutes a central reference for perception of the environment in that it provides meaning for otherwise meaningless sensations.

6. This is also acknowledged in the original study of Botvinick and Cohen (1998).

7. For example, in many studies, the onset of involuntary actions was unpredictable, and the onset of voluntary actions were predictable. Thus, temporal predictability rather than actors’ intentions can explain the differences between temporal binding in voluntary and involuntary conditions.

8. In this study, about 70% of the binding effect observed with active movements were present in conditions with passive movements.

9. Remember, for example, that both, interoception and exteroception are combined in the perceived size of an object held in the hand, whereas their relative impact varies depending on context conditions.

10. At least when the questionary data are considered. Here, the real hand appears to be at the same location as the fake hand after synchronous tactile stimulation of both.

11. Features denote perceptual properties of an event, which often closely match with simple physical properties, such as shape, locations, or color (see e.g. Hommel, 2004; Treisman, 1996).
